# Old but gold: Is the Judet procedure still a viable option for posttraumatic knee stiffness in 2024? A comprehensive systematic review and meta‐analysis

**DOI:** 10.1002/jeo2.70079

**Published:** 2024-11-07

**Authors:** Vito Gaetano Rinaldi, Iacopo Sassoli, Alberto Fogacci, Antongiulio Favero, Giada Lullini, Massimiliano Mosca, Mattia Morri, Stefano Zaffagnini, Giulio Maria Marcheggiani Muccioli

**Affiliations:** ^1^ II Clinica Ortopedica e Traumatologica, IRCCS Istituto Ortopedico Rizzoli Bologna Italy; ^2^ UOC Medicina Riabilitativa e Neuroriabilitazione, IRCCS Istituto delle Scienze neurologiche Bologna Italy; ^3^ Servizio di Assistenza Infermieristica, Tecnica e della Riabilitazione IRCCS Istituto Ortopedico Rizzoli Bologna Italy

**Keywords:** Judet quadricepsplasty, knee injury, posttraumatic knee extension contracture, range of motion

## Abstract

**Background:**

Posttraumatic extension contracture of the knee (PECK) is common after knee injury. Initial management is conservative to improve the range of motion; if it fails, surgery may be necessary. This systematic review analyses existing literature on Judet quadricepsplasty for PECK. We will assess clinical outcomes, complications, patient satisfaction and factors that may influence its success.

**Methods:**

A search was conducted on 25 November 2023, adhering to preferred reporting items for systematic reviews and meta‐analyses guidelines. PubMed, Embase and Google Scholar were used. Search strings were ([Judet] OR [quadricepsplasty]) AND (knee) AND (stiffness) and ([Judet] OR [quadricepsplasty]) AND (knee). Inclusion criteria: English articles focused on PECK, published between 2003 and 2023, and a minimum follow‐up of 24 months. Exclusion criteria: case reports, alternative techniques, knee stiffness cases not only due to trauma, a sample size of <10 patients and articles not reporting functional outcomes.

**Results:**

Among selected studies, 239 patients were considered. The average time between injury and Judet was 27 months. The population was predominantly male; the mean follow‐up was 33 months. An average intraoperative knee range of motion improvement of 79.1 degrees (confidence interval 76.9; 81.3) compared to the average preoperative starting value of 30.7 degrees was observed. This improvement decreased by 13.5 degrees at the first postoperative check and by an additional 2.4 degrees at the follow‐up, while maintaining an average value of bending above 90 degrees.

**Conclusion:**

Judet quadricepsplasty appears an effective technique for the management of PECK. The heterogeneity of included studies and the absence of standardized outcome measures limit the ability to draw definitive conclusions.

**Level of Evidence:**

Level III.

**Abbreviations**
CRPS: complex regional pain syndrome
HSS: hospital for special surgery
KSS: Knee Society Score
MINORS: methodological index for nonrandomized studies
MUA: manipulation under anaesthesia
PECK: posttraumatic extension contracture of the knee
PRISMA: preferred reporting items for systematic reviews and meta‐analyses
ROM: range of motion
STROBE: strengthening, the reporting of observational studies in epidemiology

## INTRODUCTION

Posttraumatic extension contracture of the knee (PECK) is a common complication following a knee injury. It is characterized by a decreased range of motion (ROM), pain and discomfort in the affected knee joint [29]. A variety of factors can cause PECK: first of all, inflammation and scar tissue [14].

The underlying inflammatory condition leads to scar tissue formation, which, when combined with immobilization, produces progressive knee stiffness.

Furthermore, prolonged immobilization leads to muscle weakness resulting in decreased mobility and stiffness.

All these conditions lead to a limited ROM that can make several simple patients' daily routine activities difficult [16].

This kind of stiffness has two components:

- Intra‐articular: Due to tissue remodelling resulting in the development of intra‐articular adhesions, excessive proliferation and consequent hypertrophy of fibrous scar tissue. In particular, during the healing process, initial Type III collagen (which is more pliable) is gradually replaced by Type I collagen (which is stronger but less elastic). Moreover, in normal tissue, collagen fibres are aligned in parallel, allowing flexibility and strength. In scar tissue, collagen fibres are often disorganized and cross‐linked, which increases stiffness and reduces elasticity. Furthermore, persistent inflammation stimulates fibroblast activity, leading to capsular thickening and fibrosis. This fibrotic capsule is less extensible, thereby limiting ROM [29].
- Extra‐articular: Due to the formation of adhesions between the quadriceps muscle and the bone callus, due to posttraumatic osteoreparative phenomena, femoral aponeurosis and the intermuscular septum, muscle retraction due to scar tissue and finally to skin adhesions in deeper layers.

It is important to determine the source of the stiffness, as this information will determine which procedures should be performed. Adhesion and bone impingement are the most common causes of knee stiffness after trauma. In all cases, any fractures must be healed before release can be performed, thus, a 3–6 month waiting period is required. It is important to make a compromise between managing stiffness and obtaining bone union. Moreover, it is important to identify a possible Complex Regional Pain Syndrome (CRPS). However, it is difficult to distinguish between posttraumatic stiffness and CRPS since these two conditions are often interlinked. If this condition does not revolve spontaneously, surgery should be procrastinated, waiting for the quiet phase [5].

Posttraumatic knee extension contracture of the knee can initially be treated with conservative therapy. The first therapeutic option is physical therapy, medications like nonsteroidal anti‐inflammatory drugs and corticosteroid injections [15].

While nonsurgical treatments can sometimes be effective, surgical interventions are often necessary to restore function and relieve pain [5].

Historically, many surgical options have been proposed for posttraumatic knee stiffness [5, 6, 8, 9, 11, 21, 23, 26, 27, 28].

Arthroscopic lysis of adhesions is a minimally invasive surgical procedure that can be effective in cases where the stiffness is due to novel intra‐articular scar tissue formation [1, 23]. This has become a standard technique that may be considered three months after the injury event, and sometimes even earlier if the ROM shows no further improvement, and there are no signs of active CRPS. However, it is crucial to emphasize that the fractures must have completely healed [5].

On the other hand, manipulation under anaesthesia (MUA) allows the surgeon to break up adhesions and scar tissue inside and outside the joint, limiting the ROM. This procedure is often performed as a first‐line treatment for insidious posttraumatic knee stiffness [9, 22].

Although the MUA has seen a reduction in indication in recent years as its potential risks such as fracture, failure of fixation construct, tendon rupture and cartilage damage; nevertheless, gentle manipulations can be an option before 3 months; for example, after IM nailing of an isolated femoral shaft fracture with radiological signs of union, because the contractures are not yet severe and the risk of fixation failure is low [5, 7].

Furthermore, open surgical release is typically reserved for cases where arthroscopic lysis of adhesions and MUA have not been successful. During the last century, many open surgical approaches have been proposed [3, 6, 15, 18, 19, 20].

In particular, the Judet quadricepsplasty was first described in the 1950s by the French orthopaedic surgeon Jacques Judet [10]. It involves a series of incisions and releases of the soft tissue around the knee joint, allowing the surgeon to access and manipulate the quadriceps and its tendon.

This surgical procedure comprises the release of the quadriceps tendon and surrounding soft tissues, which can become tight or contracted due to injury or prolonged immobilization.

While the Judet quadricepsplasty can effectively restore knee function, it is not without risks and potential complications. These may include infection, bleeding, nerve, tendons and muscle damage or residual joint stiffness. The decision to undergo this surgery should be carefully considered, weighing the potential benefits against the risks and recovery time.

This systematic review aimed to analyze the existing literature on Judet quadricepsplasty for PECK, including observational studies. We will assess the clinical outcomes, complications and patient satisfaction associated with this technique and the factors that may influence its success. Ultimately, this review will help clinicians make informed decisions about using Judet quadricepsplasty in treating knee contracture.

According to the authors' current knowledge, this study represents the first comprehensive review to examine the full spectrum of cases published on the Judet quadricepsplasty technique, thus filling a significant gap in the existing scientific literature.

## POSSIBLE SURGICAL TECHNIQUE

### Standard procedure Judet quadricepsplasty

**Figure 1:**
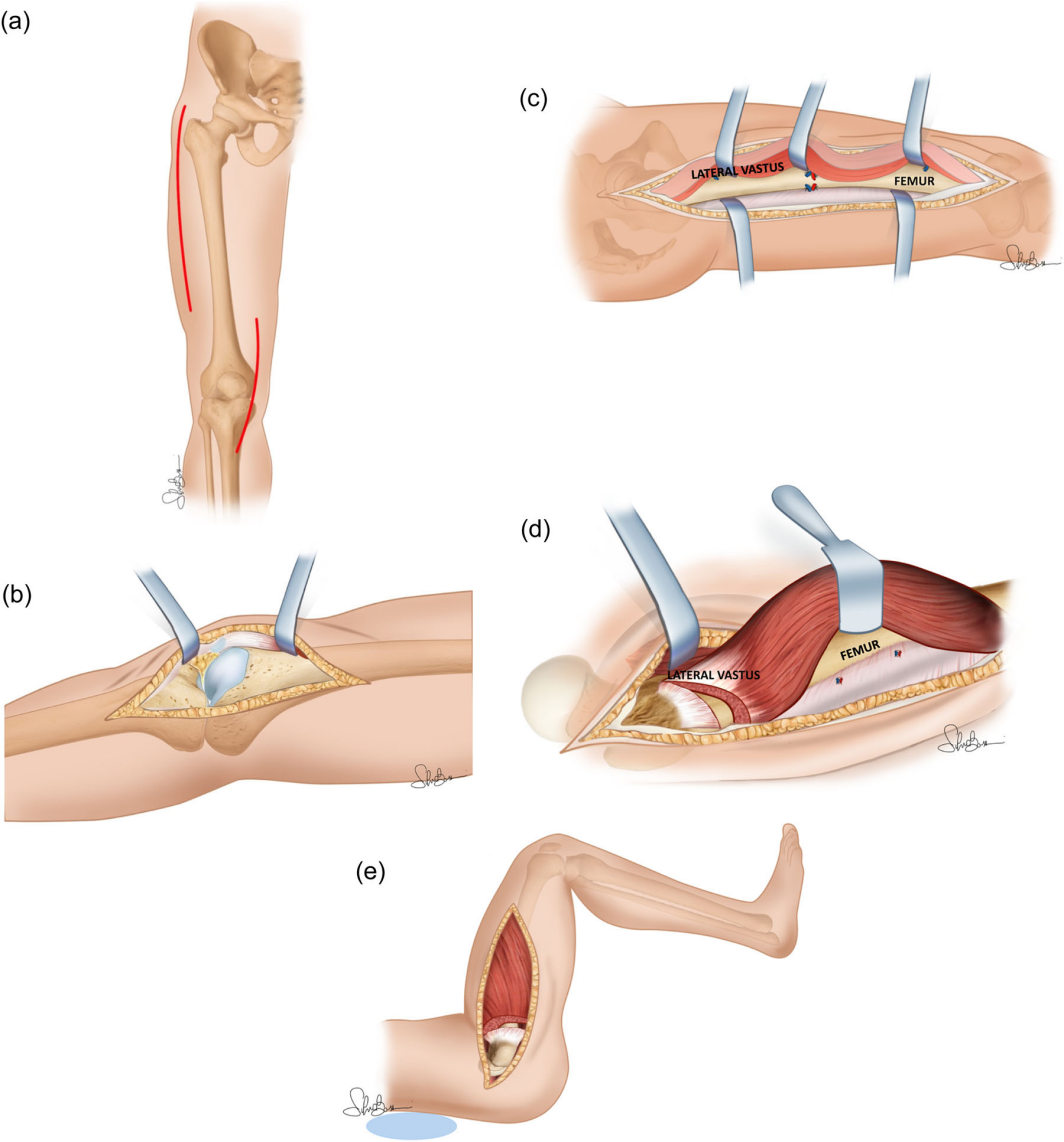
Judet quadricepsplasty. (a) Dual surgical access both medial for arthrolysis and lateral for arthromyolysis. (b) Medial intra‐articular knee approach in according to Gernez for arthrolysis. (c) Release of adhesions between anatomical layers; release of the vastus lateralis and extra periosteal disconnection of the vastus intermedius from the anterolateral cortical of the femoral diaphysis. (d) The tendinous origin of the vastus lateralis is cut completely at the level of the subtrochanteric crest and the anterior aspect of the coxofemoral capsule. (e) Knee and hip flexion causes the vastus lateralis to descend. EMC, encyclopedie medico chirurgicale.

The procedure consists of two steps: the first one is a medial parapatellar incision extending to the medial side of the tibial tuberosity. This permits access to the patellar tendon, releasing the medial retinaculum, suprapatellar pouch and intra‐articular adhesions. The second step is a long lateral incision made from the lateral aspect of the lower pole of the patella to 5 cm distal to the greater trochanter. Through the distal part of this incision, the patella and lateral retinacular tissues are freed ensuring that the patella may be easily lifted off the femoral condyles. This incision permits adhesion release and frees the vastus lateralis from the linea aspera. The vastus intermedius then is lifted extraperiosteally from the lateral and anterior surfaces of the femur. In most instances, this muscle is fibrotic and requires resection. Cautious debulking of redundant bone within the fracture callus may be done at this stage. The last step of this procedure is a proximal release of the vastus lateralis at its origin from the greater trochanter, and if necessary, the rectus femoris and the sartorius from their iliac origins, with care being taken to protect the femoral nerve (Figure 1) [25].

The two main points of the standard classical technique:

– Double open surgical access, medial parapatellar and at lateral femoral
– Detachment of the vastus lateralis origin

### Modified Judet's quadricepsplasty

**Figure 2:**
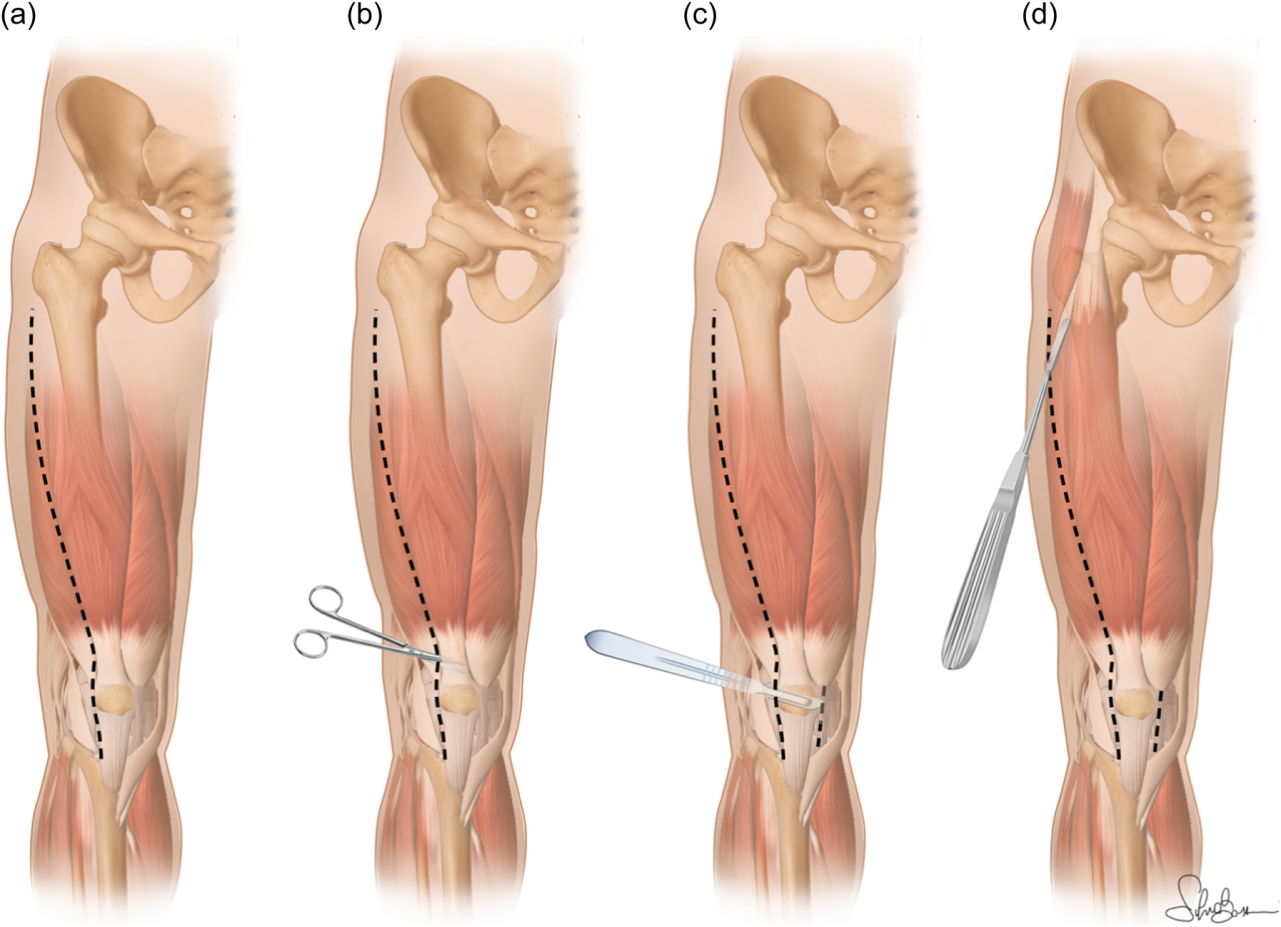
Modified Judet quadricepsplasty. (a) Illustration of the anterolateral parapatellar single open access (+possible medial mini‐open) and its proximal extension to the greater trochanter. (b) Release of the lateral retinaculum and release of the adhesions in the suprapatellar gutter and between the patella and the femoral condyles. (c) Medial release performed through the lateral approach. Vastus intermedius is also released and lifted off the anterior and lateral surfaces of the femur extraperiosteally. (d) Vastus lateralis is detached from the linea aspera until the level of the greater trochanter with a periosteal elevator without detaching its origin from the great trochanter.

The modified Judet is performed in two steps. First, a lateral incision at the knee joint begins at the tibial tubercle, extends to the proximal end through the lateral patella and ends at the third distal of the femur. The adhesion is released between the lateral patellar retinaculum and lateral femoral condyle. The medial retinaculum is dissociated from the medial femoral condyle through the same incision. If the medial lysis is incomplete, a short incision is made in the medial patellar region. Second, the extraarticular adhesions between the quadriceps femoris and the femur are released. The completion of these two lysis steps results in improving knee flexion in most patients. Even if the knee flexion is not markedly improved, the third step of conventional Judet's quadricepsplasty is not performed. A knee flexion of <90° after the two lysis steps indicates that contracture of the quadriceps femoris is affecting the knee flexion; thus, patellar traction is performed to lengthen the quadriceps femoris. After knee arthrolysis, patellar traction is performed via one of the following two methods in all patients. (a) Traction using two Kirschner wires: one through the patella, and another one through the femoral condyle. The direction of patellar traction is parallel to the femoral shaft. The direction of femoral condylar traction is vertically upward, and the traction weight is based on the criterion that the heel of the affected limb was lifted above the bed. (b) Traction using one single Kirschner wire through the patella. The traction direction is vertically upward, and the traction height is based on the criterion that the heel of the affected limb was lifted above the bed. Traction is usually initiated on postoperative day one and maintained until the knee ROM exceeds 90° (Figure 2) [28].

The two main points of the modified Judet technique:

‐ Lateral single open access (**+**possible medial mini‐open).
‐ Sparing of the vastus lateralis origin.
‐ Possible stretching of the quadriceps femoris muscle by traction if previous steps fail.

### Minimally invasive technique

**Figure 3:**
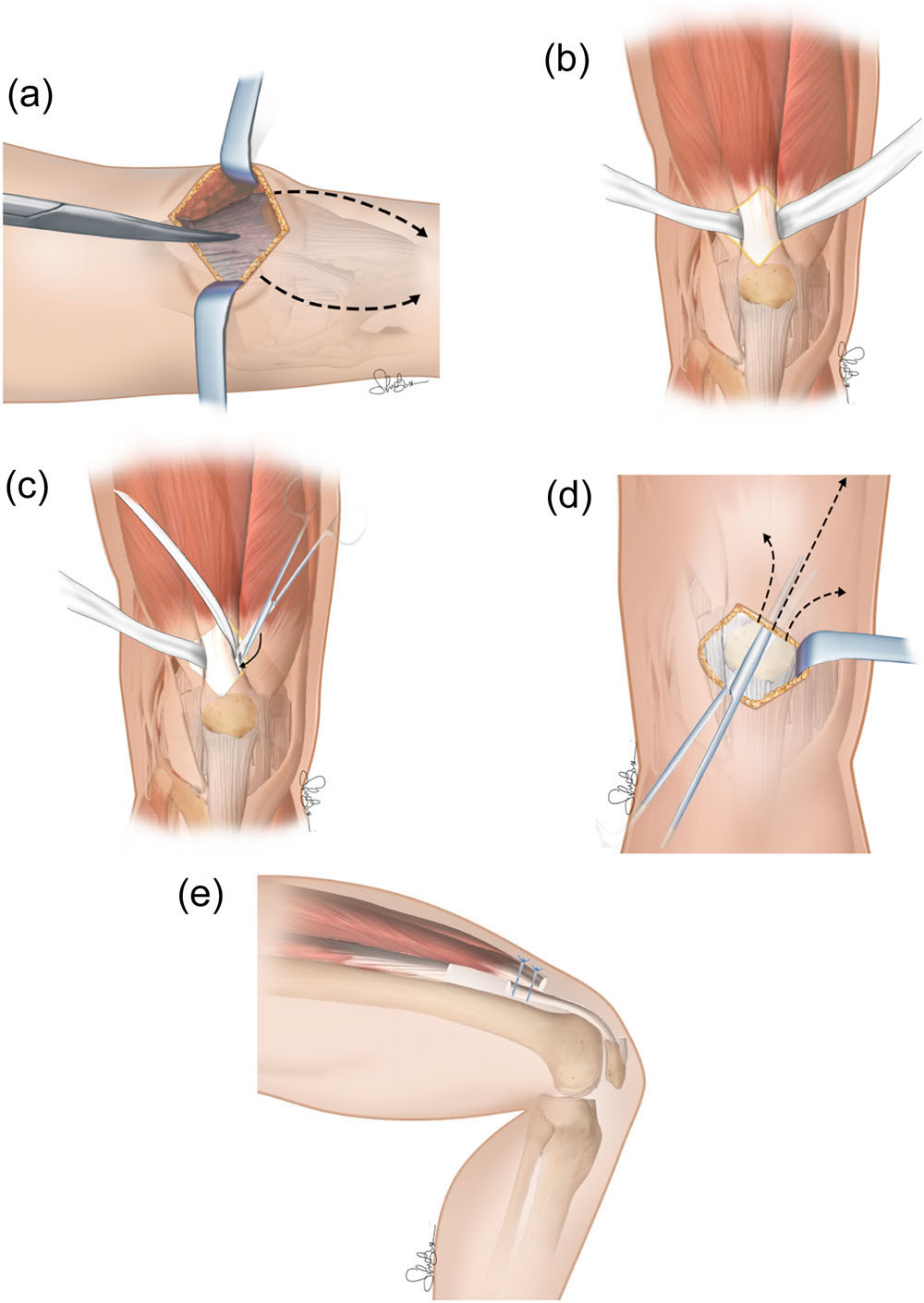
Minimally invasive quadricepsplasty. (a) Median incision proximal at the superior patellar pole and following parapatellar lateral and medial arthrotomy. (b) Rectus femoris tendon isolation. (c) Vastus intermedius tendon resected near patellar insertion. (d) Release of subcutaneous adhesions at anterolateral thigh. (e) Possible variation: a zeta plastic execution. The proximal portion of the vastus intermedius tendon could be sutured with the distal portion of the rectus femoris tendon allowing the lengthening of the extensor apparatus.

A 6–8 cm incision is made in the longitudinal median of the upper edge of the patella. The soft tissue is dissected out at both sides with a scalpel, and the rectus femoris tendon is revealed. The edge of the rectus tendon is sliced on its junction with the vastus lateralis and vastus medialis on both sides with a scalpel. The rectus femoris tendon is separated from the vastus intermedius muscle with haemostatic forceps. Attention should be paid to maintaining the smoothness and integrity of the tendon edge of rectus femoris. The rectus femoris tendon is then lifted to the upper edge of the patella with gauze. The deep surface of the rectus femoris to the ventral junction of the tendon, or an even deeper place, was bluntly separated by fingers toward the proximal part. Make sure that the fingers can slide smoothly between the rectus femoris and the vastus intermedius, without any residual adhesions. An electrosurgical knife is used to cut off the vastus intermedius tendon at the patellar attachment. Then the incompact broken ends of the vastus internus are lifted with vessel forceps and repaired with surgical scissors or an electric knife. A 3–5 cm incision is made on the quadriceps dilatation symmetrically along the upper edge of the patella on both sides by using surgical scissors. The patella is lifted, and tissue scissors are used to loosen the adhesive band in the patellofemoral joint. Buckle the knee to 110 and 120 with a smooth and progressive power gradually to avoid the self‐tear of adhesion within the tibiofemoral joint during this process; release and flexion should be performed alternately. Sharp dissection with surgical scissors is needed if fibrous adhesions still exist within the joint. To prevent the occurrence of fracture or ligament rupture, any excessive force is avoided in this process. If the resistance is too high when bending the knees, the above exploration and release process should be repeated (Figure 3) [11].

## MATERIALS AND METHODS

### Literature search

On 25 November 2023, a literature search was conducted, adhering to the preferred reporting items for systematic reviews and meta‐analyses (PRISMA) guidelines. PubMed (Medline), Embase and Google Scholar were the three online databases used for the search, and it was independently performed by two authors (I. S. and A. F.). The search strings employed were ([Judet] OR [quadricepsplasty]) AND (knee) AND (stiffness), and ([Judet] OR [quadricepsplasty]) AND (knee).

Two authors independently reviewed the titles and abstracts of the identified records. For cases where there was disagreement regarding inclusion or exclusion, a third experienced author (V. G. R.) was consulted to make the final decision. After finalizing the selection, a single author extracted the results from the full‐text articles that had been agreed upon. The evaluators remained blinded to each other's determinations throughout the process. The protocol for this systematic review was registered with PROSPERO, the international prospective register of systematic reviews, under the registration number CRD42023482102.

### Eligibility criteria

The systematic review included studies reporting clinical outcomes of posttraumatic knee stiffness treatment using arthromyolysis as per the Judet technique. Additionally, studies that met the predefined inclusion criteria were also included.

All selected articles conformed to the PICOS criteria for systematic reviews.

Our inclusion criteria for the study were as follows: (1) Articles in English, (2) Articles published between 1 January 2003 and 1 November 2023, (3) Articles with a minimum patient follow‐up of 24 months, (4) Studies focusing on posttraumatic knee (Fractures around the knee joint, including distal femur fractures and proximal tibia fractures, ligamentous injuries stiffness, complex knee injuries) cases and (5) Studies analyzing a cohort of at least ten patients.

We have excluded from our review: (1) Case reports, (2) Studies featuring alternative techniques to arthromyolysis, (3) Articles that covered knee stiffness cases that were not solely due to trauma, (4) Studies with a sample size of <10 patients and (5) Articles not reporting functional outcomes.

### Data extraction

Data extraction was performed from full‐text articles utilizing a standardized data collection form. The collected study data encompassed various key elements, such as the year of publication, clinical study type, country of origin, level of evidence, study period, inclusion and exclusion criteria, sample size, demographic information of the participants, patients' age, average time between injury and the Judet procedure, length of follow‐up and the diverse clinical outcomes and complications reported in each study.

The primary focus of the study was to evaluate the achieved flexion grade after the Judet procedure. The surgical outcomes were assessed based on Judet's criteria and the level of achieved ROM.

### Quality assessment

Two authors independently evaluated the quality and rigour of the included studies using the methodological index for nonrandomized studies (Table 1) [25]. For noncomparative studies, the ideal global score was 16, and for comparative studies, it was 24. The items were scored as follows: 0 (not reported), 1 (reported but inadequate) or 2 (reported and adequate). Consensus was achieved when both authors agreed on an item.

**Table 1:** Quality and rigor evaluation of the included studies using the MINORS.

	Ali et al. [25]	Massè et al. [29]	Alici et al. [30]	Gomes et al. [19]	Lee et al. [13]	Oliveira et al. [26]	Mahran et al. [20]	Zubairi et al. [27]	Xing et al. [11]	Bidoolegui et al. [21]	Shen et al.[28]	Luo et al. [23]	Mittal et al. [24]
**A clearly stated aim**	2	2	2	2	2	2	2	2	2	2	2	2	2
**Inclusion of consecutive patients**	2	2	2	2	2	2	2	2	2	2	2	2	2
**Prospective collection of data**	2	2	2	2	2	2	2	2	2	2	2	2	2
**Endpoints appropriate to the aim of the study**	2	2	2	2	2	2	2	2	2	2	2	2	2
**Unbiased assessment of the study endpoint**	0	0	0	0	0	0	0	0	0	0	0	0	0
**Follow‐up period appropriate to the aim of the study**	2	2	2	2	2	2	2	2	1	2	1	2	2
**Loss to follow up <5%**	2	1	2	2	2	2	2	2	2	2	2	2	2
**Prospective calculation of the study size**	2	2	2	2	2	2	2	2	2	2	2	2	2
**An adequate control group**	0	0	0	0	0	0	0	2	2	0	0	0	0
**Contemporary groups**	0	0	0	0	0	0	0	2	2	0	0	0	0
**Baseline equivalence of groups**	0	0	0	0	0	0	0	2	2	0	0	0	0
**Adequate statistical analyses**	0	0	0	0	0	0	0	2	2	0	0	0	0
**TOTAL**	14	13	14	14	14	14	14	22	21	14	13	14	14

Abbreviation: MINORS, methodological index for nonrandomized studies.

In cases where there was disagreement between the two authors regarding the inclusion or exclusion of an article, a third author with more experience intervened to make the final decision.

### Risk of bias

To assess the quality of the included studies and their risk of bias, the Strengthening the Reporting of Observational studies in Epidemiology (STROBE) checklist criteria were employed. These criteria covered aspects such as bias due to confounding, participant selection, classification of interventions, deviations from intended interventions, missing data, outcome measurement and selection of reported results.

Each criterion was categorized as ‘yes’ (applicable and satisfied in the study), ‘no’ (applicable but not satisfied in the study) or ‘not applicable’ (NA, not relevant). The risk of bias judgments was categorized as ‘Low risk’, ‘Moderate risk’, ‘Serious risk’ or ‘Critical risk’.

By comparing the obtained scores among the authors, the importance and validity of each individual study were assessed. The percentage of STROBE criteria applicable to each study was determined by dividing the number of ‘yes’ scores by the total number of applicable criteria.

All studies included in the review achieved a STROBE percentage score of more than 88.3%. This result underscores the high quality and scientific relevance of the included articles. In addition, even though the selected articles cover a large period of time, there has been a strong focus on following the proper formation of a scientific article by each author.

### Statistical analysis

A descriptive summary of the data extracted from the studies was provided. Data on the measurement of knee ROM during preoperative, intraoperative, postoperative and follow‐up periods were used to carry out the respective meta‐analyses to evaluate the surgery outcome. Given the continuous nature of the outcomes considered, the mean difference was used. Data were analyzed with Review Manager (RevMan; The Cochrane Collaboration) software.

The demographic data of the patients in the included studies were collected and analyzed through Microsoft Excel, 2016 version (Microsoft Corporation).

## RESULTS

### Study population and demographics

**Figure 4:**
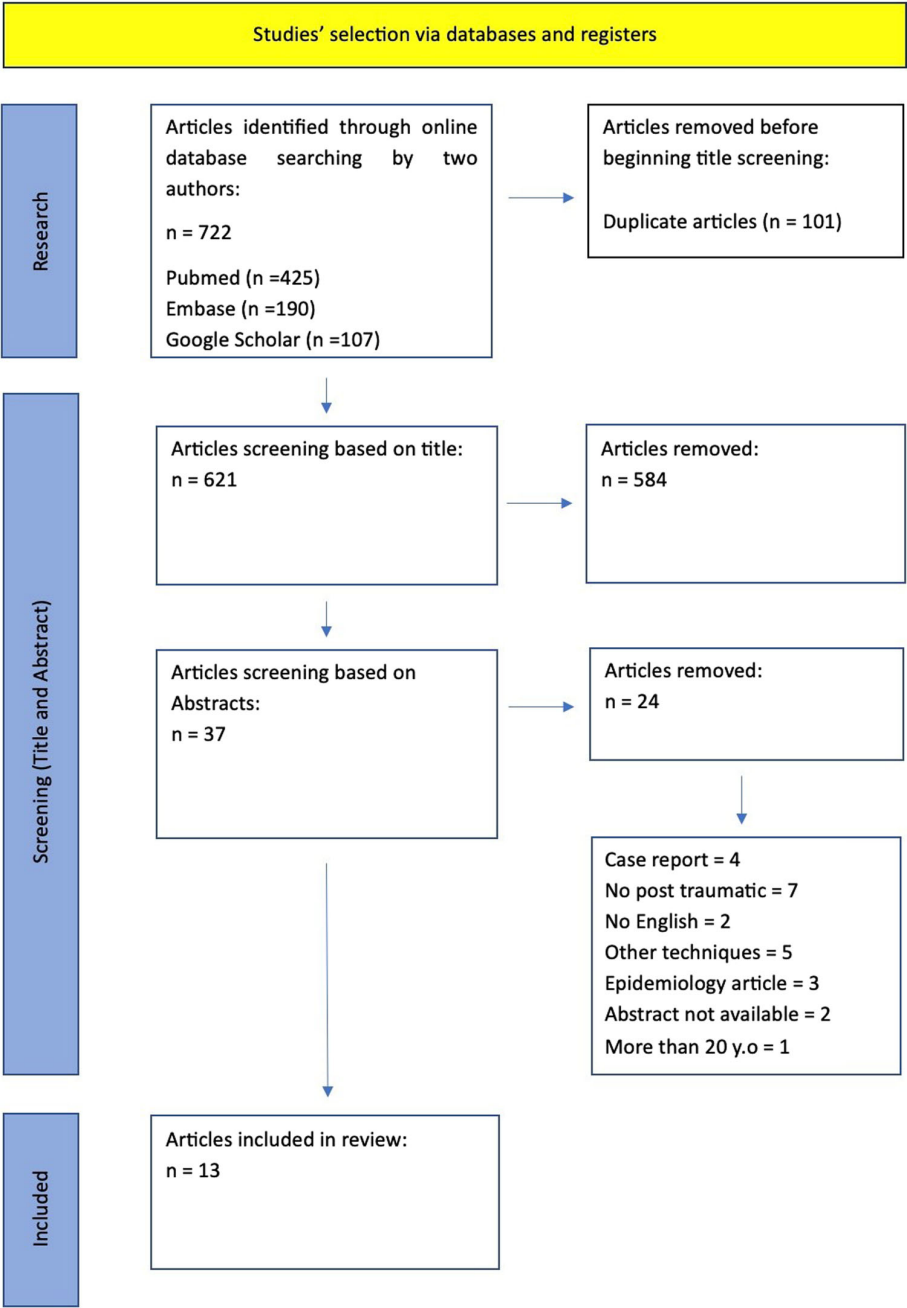
Studies selection preferred reporting items for systematic reviews and meta‐analyses flowchart.

The initial search identified 722 studies utilizing PubMed, Embase and Google Scholar as research platforms. Nonrandomized controlled trials were found. After excluding 101 duplicate studies, we were left with 621 unique studies. Subsequently, we performed screening of titles and abstracts, excluding 584 and 24 studies, respectively. Ultimately, we included 37 studies based on the inclusion/exclusion criteria outlined in our PRISMA flowchart (Figure 4). Finally, our review encompassed 13 papers.

Demographic data were provided in all of the selected studies, except for two out of the 13 where the male/female ratio was unspecified. The population characteristics considered, as illustrated in Table 2, encompassed age, sex, average follow‐up time and the duration between injury and surgery. All mean values presented were adjusted according to the population size in each study. In instances where multiple pathologies, such as limb elongation, were involved, we excluded those patients and recalculated the mean values.

**Table 2:** M/W: patients included in the study; Age‐mean: patient age included in the study; Injury‐op: time between trauma and Judet procedure.

Author	Year	N° patients	M/W	Age (years)	Injury‐op (months)	FU (months)	STROBE (%)
Ali et al.	2003	10	/	36	46	24	88.3
Massè et al.	2005	21	15/6	30	36	101	90.6
Alici et al.	2006	11	6/5	32	26	52	88.3
Gomes et al.	2009	12	6/6	30	16	/	90.6
Lee et al.	2010	10	8/2	40	40	52	88.3
Oliveira et al.	2012	45	29/16	32	/	24	90.6
Mahran et al.	2014	12	12/0	32	/	/	88.3
Zubairi et al.	2017	13	/	31	26	21	90.6
Xing et al.	2018	30	18/12	37	6	14	90.6
Bidolegui et al.	2020	11	9/2	33	/	33	88.3
Shen et al.	2020	21	18/3	36	15	18	90.6
Luo et al.	2021	15	8/7	48	56	32	88.3
Mittal et al.	2022	33	28/5	33	/	24	88.3
Med. + St. Dev.	/	TOT 244	/	34.6 ± 5.0	27.8 ± 15.8	33.4 ± 25.3	/

Abbreviations: FU, follow‐up; M/W, man/woman; STROBE, strengthening, the reporting of observational studies in epidemiology.

### Clinical results

Among the selected studies, a total of 239 patients were considered, with a mean age of 34.6 ± 5.0 years (range 30–48). The average time between injury and the Judet procedure was 27.8 ± 15.8 months (range 6–56), as reported in nine out of 13 studies. In all studies except one, the population predominantly consisted of males, with a mean percentage of men of 71% (range 50%–100%). The mean follow‐up duration was 33.4 ± 25.3 months (range 6–100).

Not all the included studies presented complete data about knee ROM obtained for the intraoperative, postoperative and last clinical follow‐up period.

Moreover, the study by Mittal et al. was excluded at the time of the execution of meta‐analysis, although it fell within the inclusion criteria because the complete data were not available.

The articles included in the study allowed the completion of three meta‐analyses comparing the mean values of knee joint excursion measured in the preoperative phase with the values of joint excursion obtained in the operating room (Table 3), during the postoperative period (Table 4) and in the last outpatient follow‐up (Table 5).

**Table 3:** A meta‐analysis comparing for each study considered the mean knee joint excursion values measured preoperatively with joint excursion values obtained in the operating room, illustrating in the right diagram the average gain between preoperative and intraoperative.

Study or subgroup	Intraoperative			Preoperative				Mean difference
	Mean	SD	Total	Mean	SD	Total	Weight (%)	IV, fixed, 95% CI
Ali	105.0	14.1	10	32.5	19.0	10	2.3	72.50 [57.84, 87.16]
Alici	111.3	12.5	11	30.0	11.5	11	4.9	81.30 [71.26, 91.34]
Bidolegui	125.4	5.1	11	36.0	6.9	11	19.2	89.40 [84.33, 94.47]
Lee	75.5	5.7	10	25.0	10.5	10	9.0	50.50 [43.10, 57.90]
Luo	107.3	7.7	15	23.3	12.3	15	9.1	84.00 [76.66, 91.34]
Mahran	116.6	13.8	19	24.5	12.5	19	7.0	92.10 [83.73, 100.47]
Masse	106.4	14.6	21	23.1	13.5	21	6.8	83.30 [74.80, 91.80]
Xing conventional surgery	109.8	9.2	30	40.0	12.6	30	15.8	69.80 [64.22, 75.38]
Xing mini‐invasive surgery	117.3	10.1	40	36.7	11.4	40	22.1	80.60 [75.88, 85.32]
Zubairi	118.9	19.3	33	36.3	27.4	33	3.8	82.60 [71.17, 94.03]
Total (95% CI)			200			200	100.0	79.10 [76.88, 81.32]

*Note*: Test for overall effect: Z = 69.85 (*p *< 0.00001). Heterogeneity: *χ*² = 97.44, *df *= 9 (*p* < 0.00001); *I*² = 91%.

Abbreviations: CI, confidence interval; *df*, degrees of freedom; SD, standard deviation.

**Table 4:** A meta‐analysis comparing for each study considered the mean knee joint excursion values measured preoperatively with joint excursion values obtained postoperative, illustrating in the right diagram the average gain between preoperative and postoperative.

Study or subgroup	Postoperative			Preoperative				Mean difference
	Mean	SD	Total	Mean	SD	Total	Weight (%)	IV, fixed, 95% CI
Bidolegui	99.0	8.8	11	36.0	6.9	11		
Bidolegui	99.0	8.8	11	36.0	6.9	11	15.2	63.00 [56.39, 69.61]
Gomes	112.0	13.0	12	10.0	9.0	12	8.3	102.00 [93.05, 110.95]
Lee	93.0	3.3	10	25.0	10.5	10	14.2	68.00 [61.18, 74.82]
Masse	79.8	18.5	21	23.1	13.5	21	6.9	56.70 [46.90, 66.50]
Oliveira	105.7	22.3	45	33.6	16.2	45	10.2	72.10 [64.05, 80.15]
Shen	92.0	3.0	21	36.0	13.0	21	20.3	56.00 [50.29, 61.71]
Xing conventional surgery	90.7	19.6	30	40.0	12.6	30	9.5	50.70 [42.36, 59.04]
Xing mini‐invasive surgery	104.8	17.9	40	36.7	11.4	40	15.3	68.10 [61.52, 74.68]
Total (95% CI)			190			190	100.0	65.62 [63.05, 68.19]

*Note*: Heterogeneity: *χ*² = 94.04, *df *= 7 (*p* < 0.00001); *I*² = 93%. Test for overall effect: *Z *= 49.97 (*p* < 0.00001).

Abbreviations: CI, confidence interval; *df*, degrees of freedom; SD, standard deviation.

**Table 5:** A meta‐analysis comparing for each study considered the mean knee joint excursion values measured preoperatively with joint excursion values obtained at the last outpatient follow‐up, illustrating in the right diagram the average gain between preoperative and last follow‐up.

Study or subgroup	Follow‐up			Preoperative				Mean difference
	Mean	SD	Total	Mean	SD	Total	Weight (%)	IV, fixed, 95% CI
Ali	87.5	13.3	10	32.5	19.0	10	4.4	55.00 [40.63, 69.37]
Alici	100.0	14.8	11	30.0	11.5	11	7.4	70.00 [58.92, 81.08]
Bidolegui	97.6	13.5	11	36.0	6.9	11	11.2	61.60 [52.64, 70.56]
Lee	94.5	5.2	10	25.0	10.5	10	17.1	69.50 [62.24, 76.76]
Luo	95.3	13.2	15	23.3	12.3	15	10.8	72.00 [62.87, 81.13]
Mahran	92.1	17.5	19	24.5	12.5	19	9.6	67.60 [57.93, 77.27]
Masse	94.8	20.9	21	23.1	13.5	21	8.0	71.70 [61.06, 82.34]
Oliveira	84.8	27.1	45	33.6	16.2	45	10.6	51.20 [41.98, 60.42]
Shen	104.0	12.0	21	36.0	13.0	21	15.8	68.00 [60.43, 75.57]
Zubairi	85.6	27.3	33	36.3	27.4	33	5.2	49.30 [36.10, 62.50]
Total (95% CI)			196			196	100.0	65.06 [62.05, 68.06]

*Note*: Heterogeneity: *χ*² = 23.36, *df *= 9 (*p* = 0.005); *I*² = 61%. Test for overall effect: *Z *= 42.46 (*p *< 0.00001).

Abbreviations: CI, confidence interval; *df*, degrees of freedom; SD, standard deviation.

In all analyses, the variation of the joint ROM showed a statistically significant improvement (*p* < 0.001) with a population of 200, 190 and 196 patients, respectively.

The intraoperative phase showed an average improvement of the ROM of 79.1 degrees (confidence interval [CI] 76.9; 81.3) compared to the average preoperative starting value of 30.7 degrees. This improvement decreased by 13.5 degrees at the first postoperative check and by an additional 2.4 degrees at the follow‐up, while maintaining an average value of bending above 90 degrees.

The three analyses all had a high value of statistical heterogeneity with an I2 ranging from 61% to 93%. To explain this condition, an additional meta‐analysis was conducted regarding the comparison between the preoperative ROM and the one reached at the last outpatient follow‐up, considering only studies in which a conventional Judet Quadricepsplasty surgical technique was applied. The meta‐analysis (Table 6) included 165 patients and confirmed an average increase of 63.2 degrees compared to the preoperative value of the joint ROM but without providing useful elements for the explanation of the statistical heterogeneity (I2 equal to 66%). Unlike, the meta‐analysis conducted compared to studies using an unconventional surgical technique showed a heterogeneity of 0% with an average increase of 68.8 degrees. This analysis included only two studies and 31 patients (Table 7).

**Table 6:** A meta‐analysis comparing the mean knee joint excursion values measured preoperatively with joint excursion values obtained at the last outpatient follow‐up, illustrating in the right diagram the average gain between preoperative and last follow‐up.

Study or subgroup	Follow‐up			Preoperative				Mean difference
	Mean	SD	Total	Mean	SD	Total	Weight (%)	IV, fixed, 95% CI
Ali	87.5	13.3	10	32.5	19.0	10	6.5	55.00 [40.63, 69.37]
Alici	100.0	14.8	11	30.0	11.5	11	10.9	70.00 [58.92, 81.08]
Bidolegui	97.6	13.5	11	36.0	6.9	11	16.7	61.60 [52.64, 70.56]
Luo	95.3	13.2	15	23.3	12.3	15	16.1	72.00 [62.87, 81.13]
Mahran	92.1	17.5	19	24.5	12.5	19	14.4	67.60 [57.93, 77.27]
Masse	94.8	20.9	21	23.1	13.5	21	11.9	71.70 [61.06, 82.34]
Oliveira	84.8	27.1	45	33.6	16.2	45	15.8	51.20 [41.98, 60.42]
Zubairi	85.6	27.3	33	36.3	27.4	33	7.7	49.30 [36.10, 62.50]
Total (95% CI)			165			165	100.0	63.24 [59.57, 66.90]
Heterogeneity: *χ*² = 20.40, *df* = 7 (*p* = 0.005); *I*² = 66%								
Test for overall effect: *Z* = 33.82 (*p* < 0.00001)								

*Note*: This meta‐analysis only considers studies describing traditional Judet quadricepsplasty.

Abbreviations: CI, confidence interval; *df*, degrees of freedom; SD, standard deviation.

**Table 7:** A meta‐analysis comparing the mean knee joint excursion values measured preoperatively with joint excursion values obtained at the last outpatient follow‐up, illustrating in the right diagram the average gain between preoperative and last follow‐up.

Study or subgroup	Follow‐up			Preoperative				Mean difference
	Mean	SD	Total	Mean	SD	Total	Weight (%)	IV, fixed, 95% CI
Lee	94.5	5.2	10	25.0	10.5	10	52.1	69.50 [62.24, 76.76]
Shen	104.0	12.0	21	36.0	13.0	21	47.9	68.00 [60.43, 75.57]
Total (95% CI)			31			31	100.0	68.78 [63.54, 74.02]

*Note*: This meta‐analysis only considers studies describing a variation of the traditional surgical technique (the traditional Judet quadricepsplasty). Test for overall effect: *Z *= 25.73 (*p* < 0.00001). Heterogeneity: *χ*² = 0.08, *df *= 1 (*p* = 0.78); *I*² = 0%.

Abbreviations: CI, confidence interval; *df*, degrees of freedom; SD, standard deviation.

According to Judet's criteria, four studies reported poor results, three reported fair results and four reported good outcomes.

Furthermore, only three out of the 13 included articles provided clinical scores. Specifically, two articles reported the Hospital for Special Surgery (HSS) score and one article reported the Knee Society Score (KSS).

## DISCUSSION

The most important finding of the present systematic review was that Judet quadricepsplasty was a surgical procedure able to guarantee an average intraoperative ROM improvement of 79.1 degrees (CI 76.9; 81.3) compared to the average preoperative starting value of 30.7 degrees. This improvement decreased by 13.5 degrees at the first postoperative check and by an additional 2.4 degrees at the follow‐up, while maintaining an average value of bending above 90 degrees, at a mean follow‐up of 33.4 ± 25.3 months (range 6–100).

Moreover, by releasing the quadriceps mechanism and lengthening soft tissues, this procedure consistently improves the knee's ROM and functional outcomes.

The reviewed studies primarily focused on long‐term outcomes of Judet quadricepsplasty in a substantial sample of PECK patients. Results demonstrated significant improvements in joint mobility and reduction in contractures, affirming the effectiveness of the procedure in restoring knee functionality.

However, variations in surgical techniques, patient selection criteria, follow‐up durations and complications pose challenges for direct comparisons.

Most of the studies included in our review examined the medium‐term outcomes of Judet quadricepsplasty in quite a large sample of patients with PECK. The results demonstrated a significant improvement in joint mobility and a reduction in contractures, confirming the efficacy of this procedure in restoring knee functionality.

In particular, the most important aspect that emerged from our review is the progressive flexion loss from the postoperative period until the end of the follow‐up.

All the included studies reported flexion loss from the immediate postoperative period until the end of the follow‐up.

In our opinion, there are several important aspects to consider regarding recovery after Judet's quadricepsplasty.

First, during the immediate postoperative period, the patients typically experience pain, swelling and limited ROM due to surgical trauma. The initial focus is on wound healing, pain management and early mobilization under the guidance of physical therapy. The pain and inflammation resulting from the surgical procedure lead to a progressive formation of adhesions and the inhibitory effect on normal movement due to pain relief [17].

On the other hand, during the follow‐up period, spanning months to years, the initially achieved improvements gradually diminish due to scar formation and the loss of tissue plasticity caused by the surgical intervention [10].

Furthermore, not all the included studies reported complications analysis and rate.

Only three of the 13 included articles report clinical scores. Specifically, two articles report HSS [8, 28] and one article reports the KSS [15].

According to the literature, the complications during or/and after quadricepsplasty were mainly injuries by knee‐extension apparatus, skin lesions, asthenia when extending knees, re‐adhesion and rare posterior femoral condyle fracture [4, 14, 24, 29].

A study conducted by Bidolegui et al. [5] examined the rates of intraoperative and postoperative complications associated with Judet quadricepsplasty. The results indicated that the procedure is generally safe, with a low incidence of major complications such as infections and neurovascular injuries.

An important aspect that emerged from our review is the duration of follow‐up in the various included studies.

Therefore, it is important to consider that many studies on Judet quadricepsplasty include short to medium‐term follow‐up, typically ranging from 6 months to 2 years [2, 5, 16, 17, 19, 20, 28, 30] while others evaluated outcomes up to several years after the procedure [3, 12, 18, 24].

This duration allows researchers to assess the initial outcomes of the procedure, monitor postoperative recovery and evaluate early improvements in ROM and functional outcomes.

However, to assess the long‐term effectiveness and durability of Judet quadricepsplasty, some studies have included longer follow‐up periods. These studies may extend their observations to 5 years, 10 years or even longer.

Certainly, longer‐term follow‐up provides valuable information on ROM maintenance, functional outcomes and patient satisfaction over an extended period.

Another debated issue in the literature is the variability in surgical techniques used in Judet quadricepsplasty. Some studies adopted a more conservative approach, focusing on the release and lengthening of the quadriceps mechanism, while others explored additional procedures such as High Tibial Osteotomy, Distal Femoral Osteotomy and Deflexion Osteotomy.

The variability in surgical approaches emphasizes the need for further research to identify the optimal method for achieving superior outcomes in knee surgery. Future studies should aim to establish standardized protocols for performing Judet quadricepsplasty. This includes defining precise surgical techniques, instrumentation and postoperative care protocols. Standardized protocols would not only enhance the comparability of study results but also contribute to refining surgical practices, ultimately advancing the field and improving patient care. Despite the positive findings regarding the effectiveness and safety of Judet quadricepsplasty, several limitations need to be considered for this systematic review. The heterogeneity among the included studies poses a significant challenge, as the variations in study designs, patient populations and surgical techniques may introduce potential biases and confounders. Additionally, the absence of standardized outcome measures across the studies further complicates the synthesis of clear results, making it challenging to draw definitive conclusions on the overall efficacy and safety of Judet quadricepsplasty. Improving standardized clinical assessment through the incorporation of ROM, Pain Scores and Functional Outcome Scores (e.g., KSS) is essential. These scores encompass both objective measures, such as knee alignment and stability, and patient‐reported outcomes, including functional activities and satisfaction. This comprehensive approach ensures a thorough evaluation of functional improvement following posttraumatic knee surgery. Additionally, integrating Patient Satisfaction and Quality of Life measures (e.g., SF‐36, EQ‐5D) further enhances the consistency and reliability of data. These standardized assessments not only facilitate meta‐analyses and systematic reviews but also strengthen the evidence base guiding clinical practice in this specialized field.

## CONCLUSIONS

The surgical technique of Judet quadricepsplasty allows to obtain a significant improvement in the knee ROM. Despite the high heterogeneity found, all studies showed significant improvement with significant clinical relevance. At the follow‐up, the patients had an average articular ROM of more than 90 degrees, although it should be noted that this improvement was less than that obtained in the operating room with a reduction trend of the articulate ROM. The postoperative phase plays an important role in joint recovery and careful monitoring of this period of treatment and adequate physiotherapy are elements that need further studies to be able to correctly evaluate the results obtained by the patient in the long term. In the field of surgical techniques, we can observe how studies that present variations from the conventional approach emerge. Such surgical modalities seem to be able to give some benefits for the patients in terms of joint excursion of the knee but such results are to be evaluated with extreme caution as they have been studied only on a very small group of patients.

The variability in surgical approaches emphasizes the need for further research to identify the optimal method for achieving superior outcomes in knee surgery. Standardized protocols would not only enhance the comparability of study results but also contribute to refining surgical practices, ultimately advancing the field and improving patient care.

However, Judet quadricepsplasty appears to be an effective and definitive surgical technique for patients with PECK, although not without risks.

## AUTHOR CONTRIBUTIONS

All authors have contributed to the development of the research questions and study design. V. G. R. and G. M. M. M. identified the method of the review protocol. I. S., A. F.(1), A. F.(2) and V. G. R. developed and conducted the search strategy and data extraction. V. G. R., G. L. and M. M. developed the first and subsequent drafts of the manuscript and G. M. M. M. and S. Z. revised it. All authors reviewed and approved the manuscript.

## CONFLICT OF INTEREST STATEMENT

The authors declare no conflict of interest.

## ETHICS STATEMENT

This systematic review and meta‐analysis were conducted in accordance with the highest ethical standards. The study involved a comprehensive analysis of previously published literature, and no new patient data were collected directly by the authors. As such, formal ethical approval was not required. All referenced studies were reviewed to ensure compliance with ethical guidelines, including informed consent and institutional review board (IRB) approval where applicable. PROSPERO registration number CRD42023482102.

## Data Availability

Data are available upon request to the corresponding author.
